# Improved Robot Path Planning Method Based on Deep Reinforcement Learning

**DOI:** 10.3390/s23125622

**Published:** 2023-06-15

**Authors:** Huiyan Han, Jiaqi Wang, Liqun Kuang, Xie Han, Hongxin Xue

**Affiliations:** 1 School of Computer Science and Technology, North University of China, Taiyuan 030051, China; sz202207005@st.nuc.edu.cn (J.W.); 20030747@nuc.edu.cn (L.K.); 19860733@nuc.edu.cn (X.H.); 20200001@nuc.edu.cn (H.X.); 2 Shanxi Key Laboratory of Machine Vision and Virtual Reality, Taiyuan 030051, China; 3 Shanxi Vision Information Processing and Intelligent Robot Engineering Research Center, Taiyuan 030051, China

**Keywords:** robot path planning, deep reinforcement learning, DDQN, expert experience

## Abstract

With the advancement of robotics, the field of path planning is currently experiencing a period of prosperity. Researchers strive to address this nonlinear problem and have achieved remarkable results through the implementation of the Deep Reinforcement Learning (DRL) algorithm DQN (Deep Q-Network). However, persistent challenges remain, including the curse of dimensionality, difficulties of model convergence and sparsity in rewards. To tackle these problems, this paper proposes an enhanced DDQN (Double DQN) path planning approach, in which the information after dimensionality reduction is fed into a two-branch network that incorporates expert knowledge and an optimized reward function to guide the training process. The data generated during the training phase are initially discretized into corresponding low-dimensional spaces. An “expert experience” module is introduced to facilitate the model’s early-stage training acceleration in the Epsilon–Greedy algorithm. To tackle navigation and obstacle avoidance separately, a dual-branch network structure is presented. We further optimize the reward function enabling intelligent agents to receive prompt feedback from the environment after performing each action. Experiments conducted in both virtual and real-world environments have demonstrated that the enhanced algorithm can accelerate model convergence, improve training stability and generate a smooth, shorter and collision-free path.

## 1. Introduction

There is an increasing emphasis on autonomous robot path planning as robots are used in more and increasingly important applications [1]. In general, robot path planning algorithms are divided into two categories: traditional methods and machine learning methods [2]. Traditional methods may require extensive calculations that are difficult to meet real-time requirements or result in locally optimal solutions that fail to produce accurate paths [3]. Due to the development and popularity of Deep Learning (DL) and Reinforcement Learning (RL), there is a tendency to solve complex problems in RL by using DL’s deep network, and more academics are concentrating on DRL, especially in high-dimensional path planning problems [4].

There have been numerous researchers who have made surprising progress in DRL-based path planning algorithms [5,6,7]. The general idea is the robot iteratively explores different behaviors in diverse situations, updates its network with reward values provided by the environment and maximizes the total reward during a single path-finding process. Jiya Yu et al. [8] investigated various DL-based computer vision methods and successfully deployed them on an embedded system for autonomous robot navigation. Keyu Wu et al. [9] showed remarkable performance in real-time paths using an DNN-based 3D path planning algorithm. In 2016, Mihai Duguleana et al. [10] proposed a method to solve autonomous robot motion and validated it in Virtual Reality (VR) and real environments, respectively. Junjie Zeng et al. [11] successfully guided a robot’s moves through continuous control signals in a dynamic environment by combining the Jump Point Search (JPS) algorithm with the asynchronous advantage Actor–Critic (A3C) algorithm. Yinliang Chen et al. [12] proposed an improved deep deterministic policy gradient (DDPG) path planning algorithm incorporating sequential linear path planning (SLP) to address the problem of robot avoidance of dynamic obstacles. In 2020, MG et al. [13] investigated the problem of multi-intelligence collaboration. Jialun Cai et al. [14] proposed a path planning method combining deep reinforcement learning and semantic information to effectively improve the autonomous decision-making capability of robots. Tomoaki Nakamura et al. [15] proposed a local path planning method in narrow environments using DQN. Mingyu Cai et al. [16] completed a continuous control of robots performing complex tasks in large-scale chaotic environments using DRL.

A classical value-based reinforcement learning algorithm, DQN [17] uses a deep neural network to approximate the Q value by feeding the model the current environment value to obtain a prediction of the reward value for each action; however, the noise of the model leads to a bias in the selection of the next action, and the greedy strategy of DQN causes problems such as overvaluation, which is solved by introducing another DQN network to separate the valuation from the selection of the action, namely DDQN [18]. However, as the dimensionality of both the environment and robot action state increases, the number of parameters required for training grows exponentially. This results in significant time and storage space consumption during training, ultimately leading to disastrous dimensions. The robot often fails to receive rewards, which poses a significant challenge for effective training. Specifically, in the context of the path planning problem, reward sparsity arises as robots receive zero rewards regardless of their actions. Furthermore, the myopic nature of the exploration strategy during early training stages poses a challenge to efficient convergence, resulting in prolonged convergence time and potential entrapment in local optima.

This paper introduces an enhanced DDQN path planning algorithm, which solves the problem of the high dimensionality of data by discretizing the input high-dimensional LiDAR data into a state space to reduce redundant information. The training process adopts a two-branch network structure, which combines expert experience with the Epsilon–Greedy algorithm to accelerate model convergence speed. Then, a well-designed reward function utilizing the “reward shaping” technique is implemented to provide timely feedback and overcome the issue of sparse rewards for the robot. Finally, we develop multiple simulation scenarios in ROS to facilitate training and testing prior to real-world implementation. The experimental results demonstrate the efficacy of our proposed method in enhancing training efficiency and generating optimal paths.

## 2. Related Work

**Figure 1:**
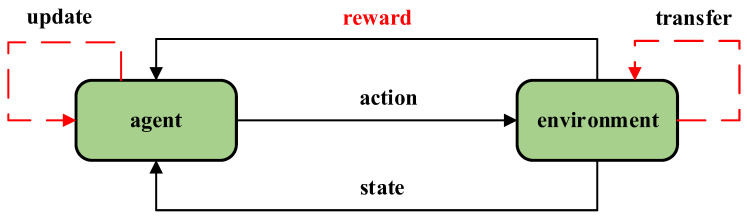
A brief process of reinforcement learning.

DRL is an artificial intelligence technique that utilizes trial–and–error iterations with the environment to acquire feedback information (reward) and optimize strategies, without relying on prior knowledge, as depicted in Figure 1.

The Markov Decision Process (MDP) [19] serves as the mathematical foundational framework and modeling tool for RL, with a particular emphasis on how the current state influences future outcomes. MDP can be represented as a quaternion <S,A,P,R>, where S denotes the state space, A denotes the action space, P represents the environmental state transition matrix and R represents the reward value provided by the environment. Moreover, it can learn online autonomously, which makes it a promising candidate for serving as a research hot for robot path planning in unfamiliar environments.

(1) $$U_{t}=R_{t}+\gamma R_{t+1}{+\gamma^{1}R}_{t+2}{+\gamma^{2}R}_{t+3}+\cdots$$

The DRL system commences with a robot’s action, and the environment transmits the state utilizing the action while providing a reward to the robot. The robot then uses the reward to update its neural network and maximizes $U_{t}$, which represents the cumulative rewards obtained throughout each round [20], as shown in Equation (1), where γ represents the discount rate (0 < γ < 1), which indicates that rewards are given less consideration as they become farther away from the present moment. Subsequently, a new action is obtained through the updated network and the next state $S_{t+1}$ is provided by the environment until the end of the round.

### 2.1. Epsilon–Greedy

To maximize the reward value $U_{t}$, the researcher utilizes the action value function $Q_{\pi }\left(s_{t},a_{t}\right)$ (shown in Equation (2)) to estimate the reward for executing strategy π after taking action $a_{t}$ in state $s_{t}$. The optimal action–value function $Q_{*}(s_{t},a_{t})$ (shown in Equation (3)) is obtained by maximizing the elimination strategy π, which represents the maximum reward for executing the optimal strategy after taking action $a_{t}$ in state $s_{t}$. The function $Q_{*}$ estimates the maximum expected cumulative reward for each action in the current state, guiding the robot to select the action with the highest expected rewards. As a result, the DRL algorithm based on value employs a deep neural network to approximate the optimal action–value function.

(2) $$Q_{\pi }\left(s_{t},a_{t}\right)=E\left[U_{t}|S_{t}=s_{t},A_{t}=a_{t}\right]$$

(3) $$Q_{*}\left(s_{t},a_{t}\right)=\underset{\pi }{\max}Q_{\pi }\left(s_{t},a_{t}\right)$$

(4) action=arg_max(Q_Net(state)),randomaction,Epsilon1−Epsilon

By employing the greedy method, the model may easily fall into a locally optimal solution. Therefore, it is advisable to encourage the model to explore more at the beginning of training rather than being restricted to selecting the action with the highest reward. During training, the possibility of the robot executing the action given by the model is Epsilon, whereas the probability of taking a random action is 1−Epsilon, as shown in Equation (4). In the initial stage, due to the small value, the robot is in a state of random exploration [21]. This approach can accelerate the convergence of the model and reduce the risk of becoming trapped in a local optimum.

### 2.2. Prioritized Experience Replay

**Figure 2:**
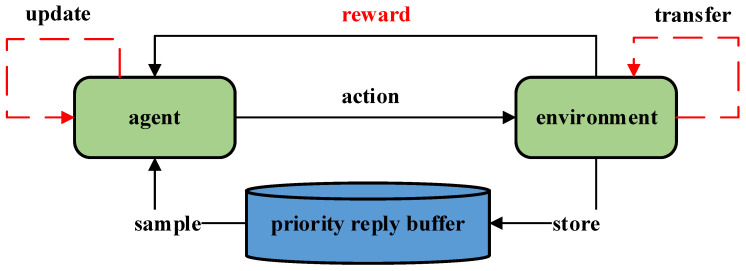
A brief process of reinforcement learning with a priority reply buffer.

During the training process, the robot–environment interaction data are typically stored in a quaternion (shown in Equation (5)) in the experience replay queue for future utilization. In the subsequent training process, a batch of data is extracted from the queue and input into the model, thereby significantly enhancing data utilization. Tom Schaul et al. [21] introduced Prioritized Experience Replay, as depicted in Figure 2.

(5) <state,action,reward,next_state>

Since each interactive data have a different impact on model enhancement, to improve the efficiency of data utilization, it is necessary to train the data that greatly improve the model. This means that model performance should be enhanced by selectively sampling data with a high TD error (representing the difference between the model output and the true value), while reducing the amount of low-quality data. To achieve this, Equations (6) and (7) were used to calculate the priority and sampling rate of each piece of data, where $\delta_{i}$ represents the TD-error value of the data; to prevent the sampling rate from being too small, ε is added. α is an indicator that regulates the effect of priority on the probability of adoption, when α equals 0 it is traditional uniform sampling, and if α equals 1 it is exactly the priority-based sampling approach.

(6) $$p_{i}=\left|\delta_{i}\right|+\varepsilon$$

(7) $$P\left(i\right)=\frac{p_{i}^{\alpha }}{\sum_{k}p_{k}^{\alpha }}$$

Since the use of the priority-based sample method is biased to the original distribution and will affect the convergence of the model, adjustments should be made in the calculation related to gradient descent. The gradient should be modified as ${\omega_{i}\cdot \delta }_{i}$ when performing backpropagation as shown in Equation (8), where β equals to 1, and then the update of the network is unbiased. In typical reinforcement learning scenarios, the unbiased nature of the updates is most important near convergence at the end of training. In practice, it is common to linearly anneal β from its initial value 0 to 1 [21].

(8) $$\omega_{i}={\left(\frac{1}{N}\cdot \frac{1}{P\left(i\right)}\right)}^{\beta }$$

**Figure 3:**
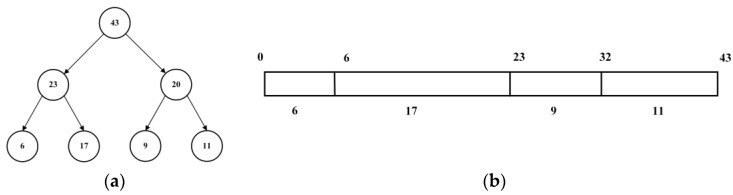
(**a**) sumTree sructure. (**b**) sumTree approximate expression.

The determination of priority p is contingent upon the TD error; however, computing p for all samples and subsequently sorting them can be prohibitively expensive [22]. To achieve a high-speed, high-priority sampling method with a high sampling rate, the “sumTree” data structure is utilized as depicted in Figure 3a. There are four leaf nodes representing the priority of each data respectively, which must be sampled according to its priority. The “sumTree” structure can be approximated as a line as shown in Figure 3b, on which a point is randomly sampled. Therefore, the “sumTree” structure does not need to sort data. The “sumTree” data structure is used to sort the improvement level of the model for each data. The data that significantly improve the model will be sampled and the goal of sampling will be realized by priority. This approach greatly enhances both the training efficiency and speed of convergence of the model in comparison to experience replay.

### 2.3. Double DQN

**Figure 4:**
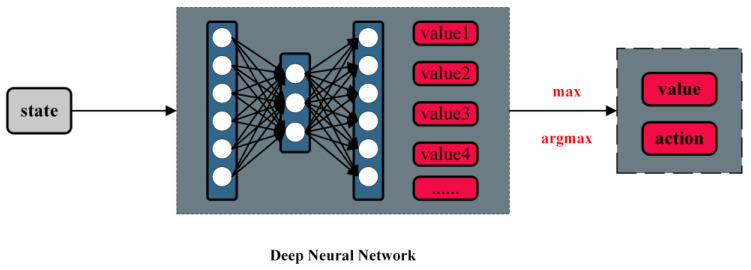
Brief structure of the DQN model.

In Figure 4, Mnih et al. [17] proposed a reinforcement learning model using deep Q networks. This network model combines neural networks and Q-learning to address the curse of dimensionality in Q-learning by replacing Q value tables with neural networks. The DQN network takes the surrounding state as input and outputs the maximum expected reward for each action after passing through the neural network. Consequently, in the current state, the model selects the action with the highest reward value as its optimal response.

**Figure 5:**
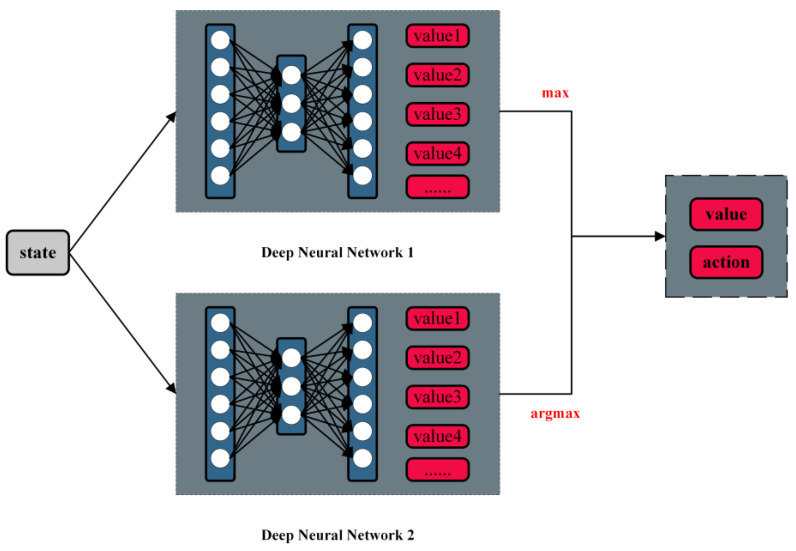
Brief structure of DDQN model.

The DQN network’s greedy strategy and the interference of noise can lead to overestimation in the model. Compared to using the same network for both maximum value judgment and estimated value calculation, Hado van Hasselt et al. [18] proposed the DDQN network (shown in Figure 5), which introduces an additional network to decouple selection from the calculation, effectively addressing overestimation issues caused by DQN.

Despite these surprising achievements, there are still several problems in implementing DRL-based path planning algorithms, some of which are listed below:

1. As the training environment becomes more complex, the number of parameters required by the model increases [3]. This may trigger a dimensional catastrophe, leading to a significant increase in computational effort ultimately extending the training time.
2. Traditional algorithms do not solve the problem of reward sparsity in path planning [23]. This is one of the reasons why DRL training is locally optimal.
3. Reinforcement learning is very sensitive to the initial values of the training model, poor initial values of the model may lead to a slow convergence rate or even to a local optimum [24].

## 3. Materials and Methods

In this paper, we present an enhanced DRL-based path planning algorithm. We first performed preprocessing to reduce the dimensionality of the data, and then fed the resulting lower-dimensional data into a new two-branch deep network for training. In addition, we introduced the combination of “expert experience” with the Epsilon–Greedy algorithm and the Prioritized Experience Replay algorithm for model training. Finally, we optimized the reward function to address the issue of reward sparsity in training. In this section, we introduce four enhancements to the traditional DDQN algorithm, namely dimensional discretization, two-branch network architecture, expert knowledge integration and reward function design.

### 3.1. Dimensional Discretization

**Figure 6:**
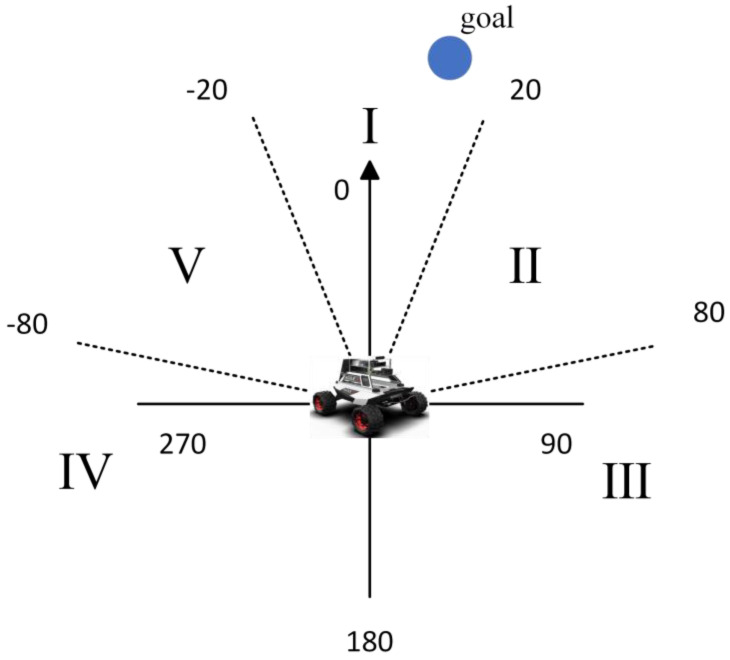
The relative position of the target point to the robot.

**Figure 7:**
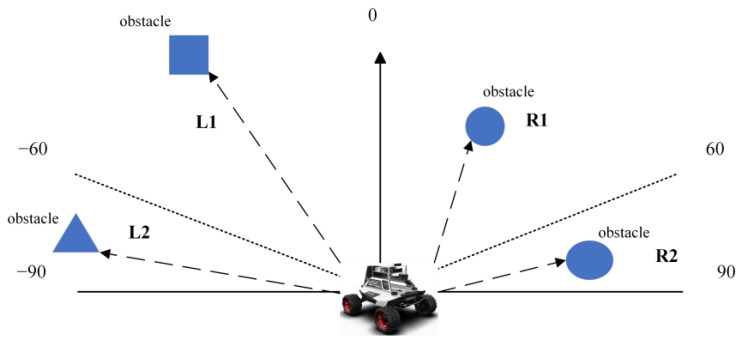
Position of the obstacle relative to the forward direction of the robot.

If unprocessed sensor-generated data are input into the network during training, it may result in issues such as slow training and challenge model convergence due to high data dimensionality, significant data correlation and increased data redundancy. The target locations associated with the robot’s forward direction were categorized into five intervals: [−20, 20), [20, 80), [80, 180), [180, −80) and [−80, −20) as shown in Table 1 and Figure 6. In the obstacle avoidance section, we only considered obstacles located within the robot’s forward direction between [–90, 90] degrees and divided them into four quadrants based on a 180 degree range, obtaining the positional information of each part from the nearest obstacle (we only consider the information of the closest obstacle), as illustrated in Figure 7. Meanwhile, the robot’s continuous operation was partitioned into five states, significantly reducing computational complexity and adhering to the robot’s movement rule as presented in Table 2.

### 3.2. Two-Branch Network Structure

**Figure 8:**
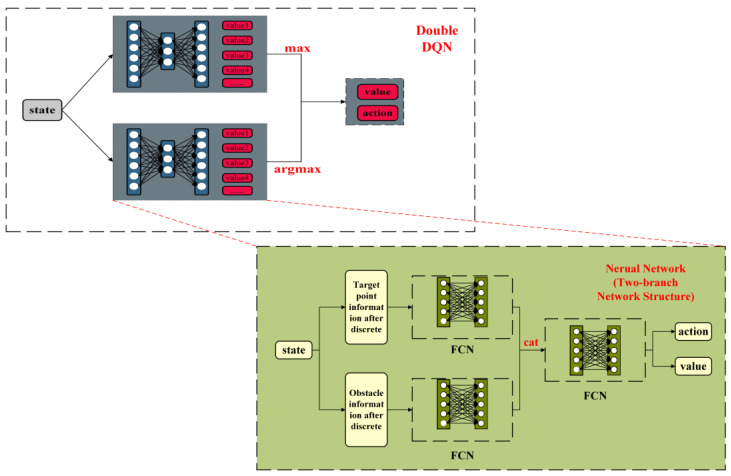
Two-branch Network Structure.

Imitating the design of DDQN, we designed a dual branch network structure on the basis of it to decouple obstacle avoidance and navigation, in which obstacle relative position and discrete target point relative position were respectively fed into the navigation module and obstacle avoidance module of the network. In the dual-branch network structure, we partitioned data into the obstacle avoidance module and the navigation module. The former requires obstacle information while the latter necessitates relative target point position. We input the four discrete values of the four distinct regions into the obstacle avoidance module and input the three parameters (the distance from the robot to the target point, its steering angle and target point position) into the navigation module. The inputs were then fed into a fully connected neural network, which generated 64 dimensions per network and a total of 128 dimensions when combined. The two-branch network was integrated separately into the fully connected neural network (FCN), and subsequently, the model output estimations for each action, as illustrated in Figure 8.

### 3.3. Expert Experience

The original Epsilon–Greedy algorithm encourages the intelligence to explore at the beginning of training and take the next step randomly to learn from experience and update the network parameters. The original Epsilon–Greedy algorithm promotes exploration during the initial stages of training, where the next step is taken randomly to facilitate experiential learning and update network parameters.

In this paper, the initial random exploration during training was replaced by the imitation of “Expert Experience” generated from discrete low-dimensional data, allowing for robot behavior to follow “expert experience” with a certain probability. “Expert experience” is not the optimal solution for path-finding; thus, its proportion gradually diminishes during training, resulting in the model being more inclined towards utilizing traditional random exploration strategies. The “Expert Experience” strategy proposed in this paper mitigated the issues of high variance and instability during the initial stages of model training.

(9) $$exp_{action}=\left\{\begin{array}{cc} \exp\_ang+\exp\_col, & -2<\exp\_ang+\exp\_col<2\\ 2, & \exp\_ang+\exp\_col\geq 2\\ -2, & \exp\_ang+\exp\_col\leq -2 \end{array}\right.$$

Discrete values assigned to the navigation and obstacle avoidance modules were based on expert experiences to guide the robot’s movement considering the relative position of the robot and the target point. Each relative position corresponded to the expert experience of only the navigation module. Similarly, the relative positions of the robot and the obstacles were considered to guide the movement of the robot, and each relative position corresponded to the expert experience of the obstacle avoidance module, as shown in Table 3 and Table 4. The expression is shown in Equation (9); thus, we can leverage the total expert experience to effectively guide the robot’s movement.

**Figure 9:**
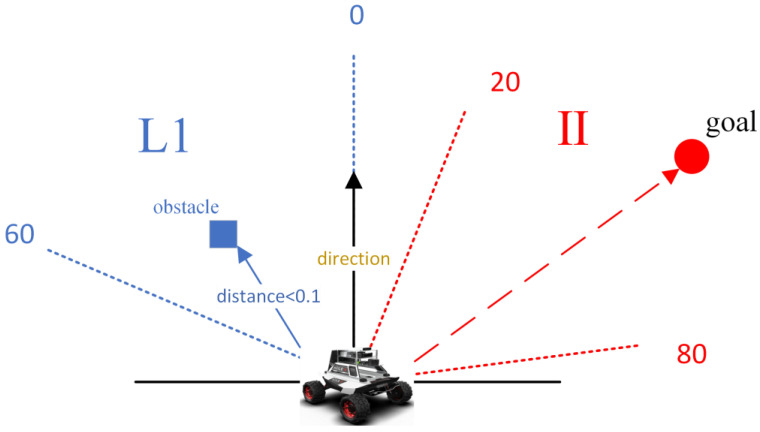
Examples of using expert experience.

As shown in Figure 9, in this case, we specified the robot forward direction to be 0 degrees, considering the obstacle avoidance module and the navigation module, respectively. Since the angle of the target point relative to the robot forward direction was between [20, 80], the expert experience of the navigation module provided “−1” to guide the motion. The nearest obstacle was located in the L1 area, and the distance was <0.1 m, so the expert experience of the obstacle avoidance module provided “−2” to guide the motion. According to Equation (9), the total sum of expert experience was “−2”. Using the expert experience, the robot motion could be guided directly, which corresponded to the discrete action value of “−2” (Turn Right with big angle).

### 3.4. Reward Function

The reward function only provides feedback on the achievement of a target point or collision with an obstacle, resulting in Sparsity of rewards and potentially leading to blind exploration, thereby complicating training. In this paper, we proposed a reward feedback system with continuous monitoring to ensure that the robot received precise and consistent rewards at each step. For many practical problems, defining a good reward function is nontrivial [25].

There are usually three methods to solve the reward sparsity problem, which are Reward Shaping [26], Intrinsic Curiosity Module (ICM) [27] and Reverse Curriculum Learning [28]. Reward Shaping guides training by designing a more reasonable reward function and ICM improves learning efficiency and performance by increasing curiosity module, while Reverse Curriculum Learning designs a reasonable training process for robots to train from easy to difficult. In this paper, Reward Shaping was used to improve the reward function.

The robot receives a reward of +300 when it reaches the target point, and a reward of −300 when it collides with an obstacle. In the noncollision and nonaccomplishment phase, we gave it a continuous reward by guiding it to become closer to the target and away from the obstacle at the same time. We divided the rewards into four parts: distance rewards, avoidance rewards, deviation angle rewards, and retention punishment, as shown in Equation (9).

The *distanceValue* is the difference of the distance between the robot and the target point in $S_{t+1}$ and $S_{t}$ stages. The longer the distance the robot moves toward the target point, the higher the reward will be; on the contrary, the higher the degree of deviation from the target point, the greater the penalty will be. At the same time, when the robot avoids the obstacle, we should also encourage it to continue to do so next time. *colValue* is the distance between the robot and the nearest obstacle in stage $S_{t+1}$ and stage $S_{t}$. The robot’s direction is also important for it to reach the target place smoothly. We hoped that the orientation of the robot would be close to the target point without considering obstacles, so *angValue* was set. We gave a fixed reward to the robot when its forward direction was close to the target point, and conversely, we gave it a fixed penalty if its forward direction was far from the target point, where the fixed penalty is equal to two. In order to reduce the effect of motion on the angle during the robot movement, we stipulated that this reward would not be considered when the angle change was less than 10 degrees.

To prevent the robot from performing a negative path planning strategy, we gave it a stalling penalty (*b*) at each step to motivate it to find a faster path planning solution.

(10) $$Reward=\left\{\begin{array}{cc} 300, & arrive\\ -300, & collide\\ k*distanceValue+\mu *colValue+\rho *angValue+b, & otherwise \end{array}\right.$$

Each part was multiplied by its weight respectively to form the sum of rewards. We adjusted the weight value respectively to balance each part to achieve a better guidance effect. After conducting multiple trials, we determined that utilizing the values of *k* = −2, μ = 0.03, ρ = 0.07 and *b* = −0.05 could facilitate rapid convergence of the model.

## 4. Results

To validate the feasibility of implementing the algorithm proposed in this paper, we initially constructed a simulation environment with randomly distributed obstacles, which measures 17 m in length and 13 m in width based on Gazebo’s simulation environment within the Robot Operating System (ROS), while leveraging NVIDIA GeForce RTX 2070 SUPER server for computational support. We used Ubuntu 20.04.5 LST and installed ROS-Noetic 1.15.15 for our simulation experiments.

The parameters for the enhanced algorithm are presented in Table 5, followed by migration of the trained model from simulation environment to real environment for testing its viability purposes.

### 4.1. Experimental Procedure

Navigation and obstacle avoidance model use radar sensors to detect surrounding obstacles. Training data are generated by controlling the movement of the robot and its interaction with the environment, which is then stored in a priority queue using the “sumTree” data structure for sampling.

The robot commences from the current coordinates, and the Gazebo environment publishes a random target point position to the parameter server. The robot retrieves goal point and radar output data from the parameter server, which are then fed into the network model that generates action instructions for controlling the robot’s movements.

**Figure 10:**
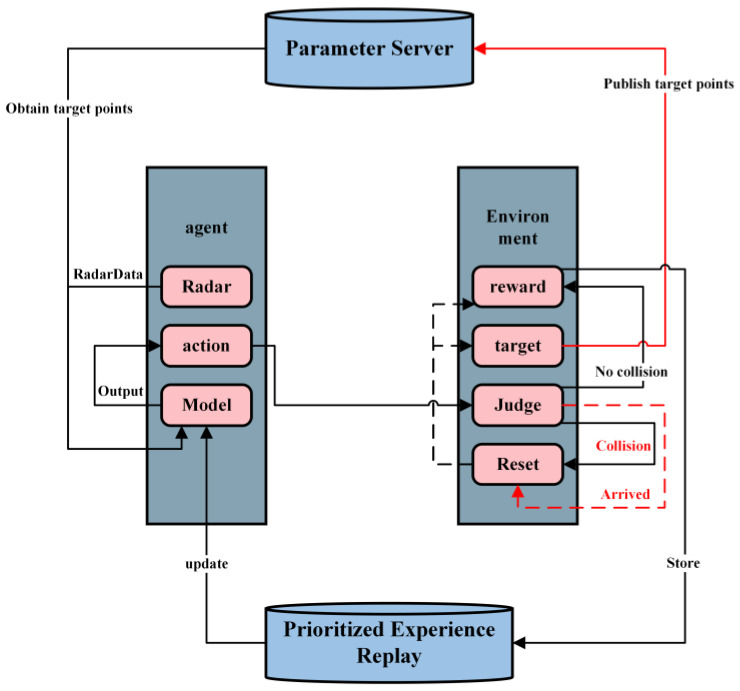
Training Process.

Subsequently, the Gazebo environment evaluates the current system state and determines whether to initiate a reset or provide an appropriate reward. Upon reaching the target point, a new one should be designated after reward issuance to commence subsequent training rounds, as depicted in Figure 10.

### 4.2. Simulation Experiments

The simulation experiments were conducted using three different training methods for comparison. Method I utilized the original deep reinforcement learning approach without any enhancements (DDQN). Method II enhanced the approach of Method I by incorporating dimensional discretization, a two-branch network and a continuous reward function into the training process. Method III was trained through the incorporation of “Expert Experience” into Method II. For each approach, we conducted separate evaluations on the episode return, success rate over nearly 100 trials, training time, and training stability.

**Figure 11:**
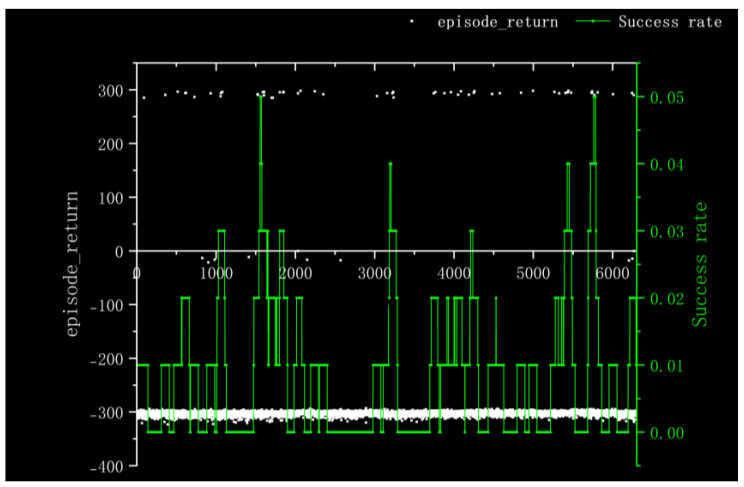
Experimental Results Obtained in Method I.

For Method I, the reward information obtained from 100 rounds of training and the success rate of nearly 100 rounds is depicted in Figure 11. It is evident that the training process is characterized by slow convergence and susceptibility to local optima, resulting in prolonged model training time. The training times were very long and it was difficult to converge the training duration for 5000 cycles in the gazebo environment with 10× acceleration reduced to 50 h. Additionally, the success rate remained at a mere 5% throughout nearly 100 rounds.

**Figure 12:**
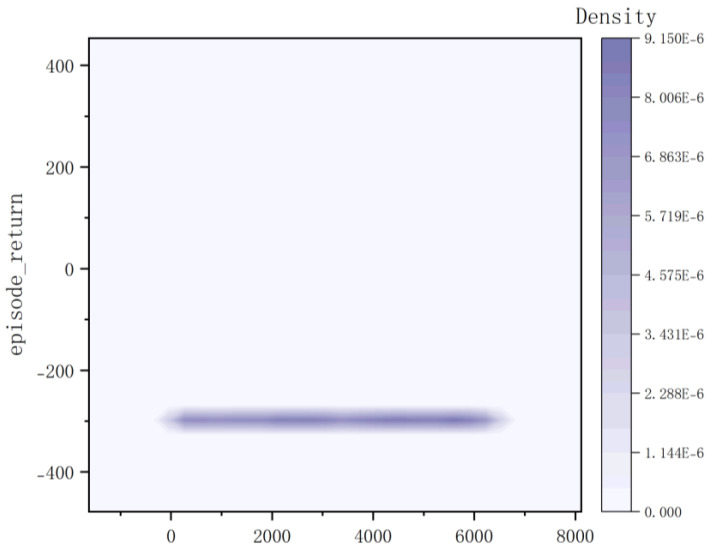
Kernel Density of Method I.

**Figure 13:**
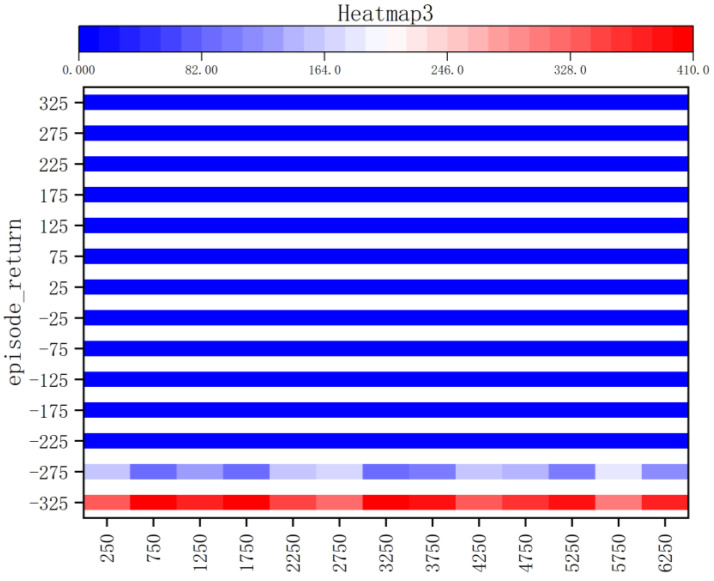
Bar Heat Map of Method I.

According to the kernel density graph in Figure 12 and the bar heat map in Figure 13, it is evident that a majority of reward values are concentrated around −300 with only a handful successfully reaching the target point, indicating an unfavorable outcome of training. The training process is notably marked by slow convergence and vulnerability to local optima, leading to extended model training duration.

Method II builds upon Method I by incorporating dimensional discretization, a two-branch network and a continuous reward function for training. This results in improved training efficacy and successful convergence of the model, enabling it to effectively navigate path planning and obstacle-avoidance tasks following a period of training.

**Figure 14:**
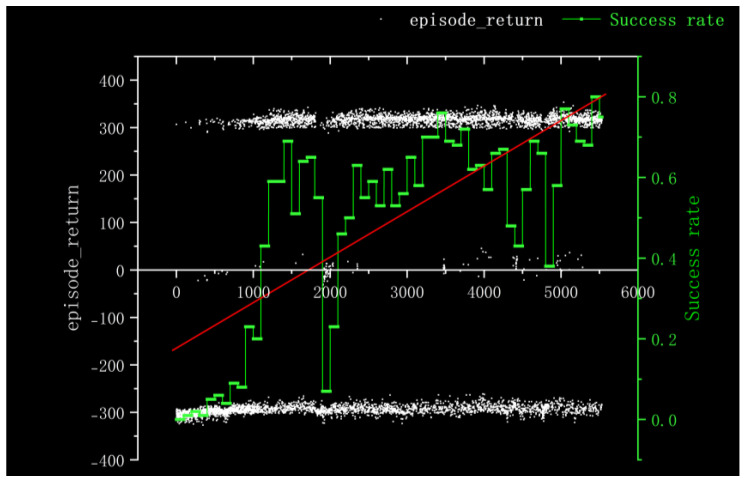
Experimental Results Obtained in Method II.

**Figure 15:**
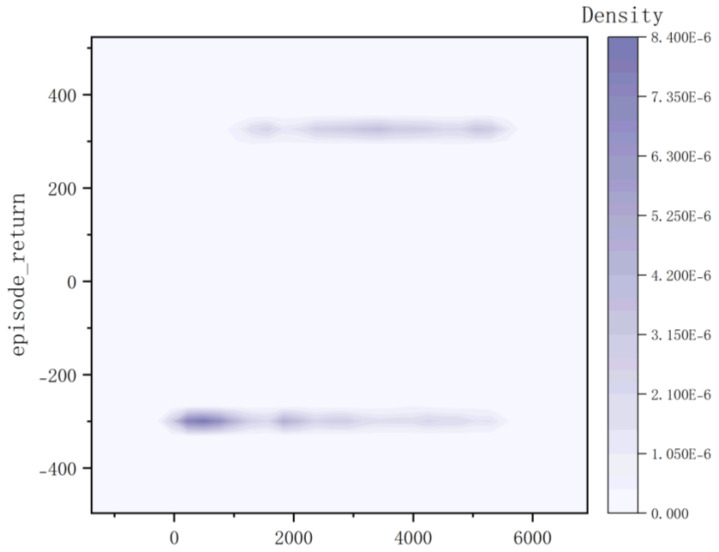
Kernel Density of Method II.

**Figure 16:**
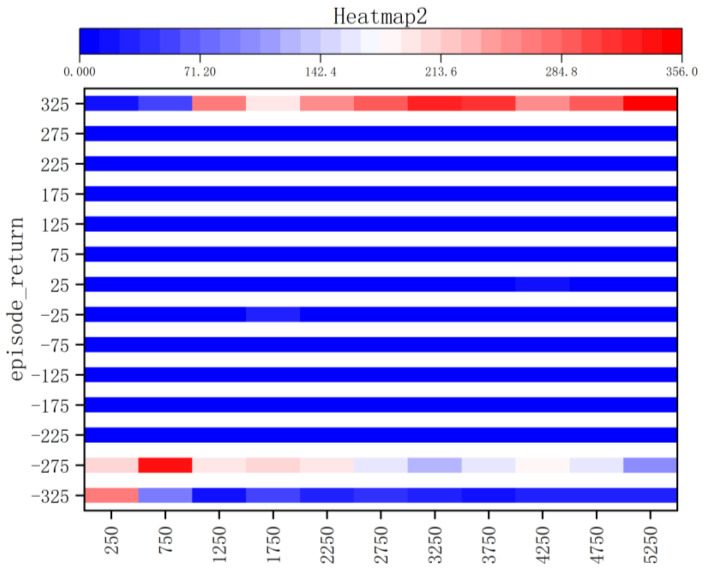
Bar Heat Map of Method II.

The reward information and average success rate obtained by Method II after training over approximately 100 rounds are illustrated in Figure 14. Due to the robot’s random exploration approach and imperfect model, its success rate is initially low during the early stages of training; however, as training progresses, the accuracy rate can reach up to 80% for almost 100 rounds. The kernel density plot and the bar heat map are shown in Figure 15 and Figure 16. It can be observed that during the early stages of training, the robot predominantly receives low reward values (−300); however, as the model progresses, there is a gradual improvement in reward distribution. At this juncture, the robot acquires obstacle-avoidance capability and successfully reaches the target point to obtain a high reward of +300. The training duration for 5000 cycles in the gazebo environment with 10× acceleration is reduced to 20 h, which enhances model stability and convergence compared to Method I.

**Figure 17:**
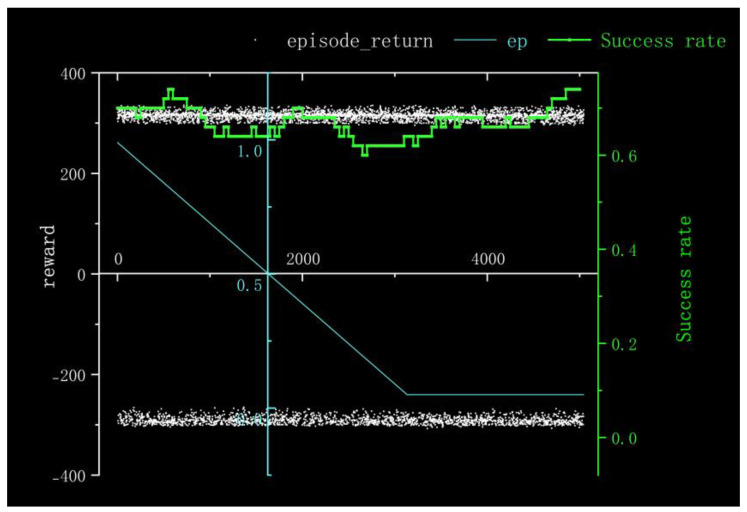
Experimental Results Obtained in Method III.

**Figure 18:**
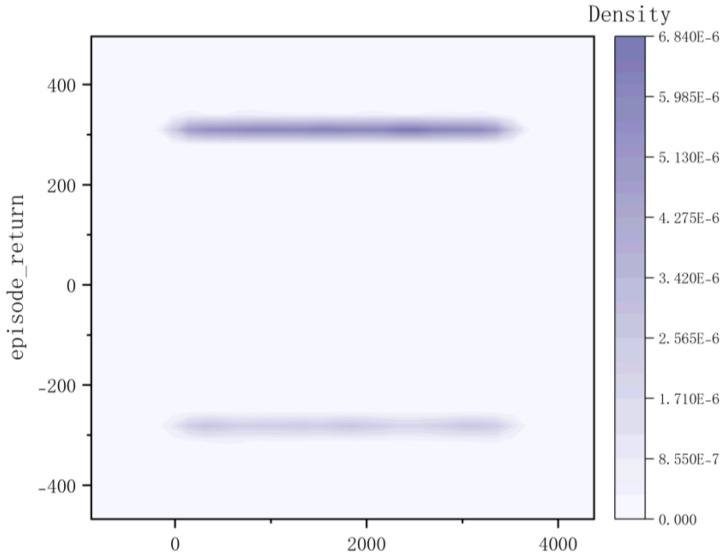
Kernel Density of Method III.

**Figure 19:**
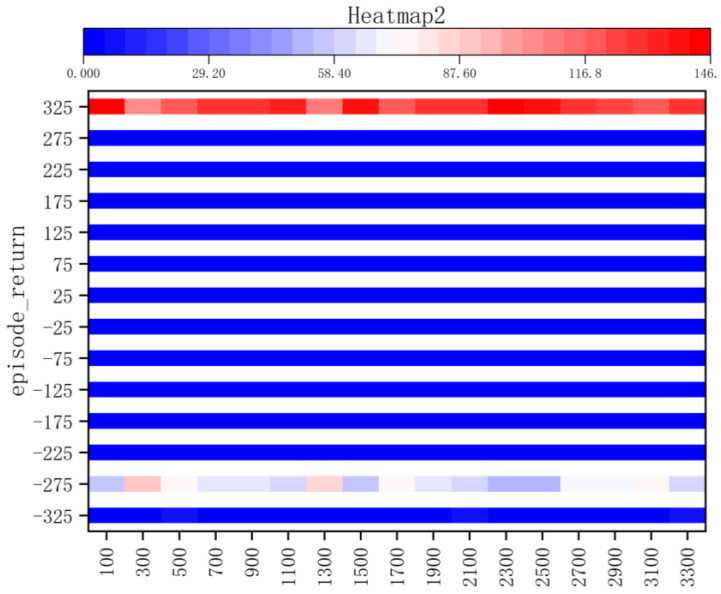
Bar Heat Map of Method III.

Figure 17 depicts the reward information and success rate of nearly 100 training rounds using Method III, where “ep” denotes the proportion of expert experience incorporated by the model. We observe an increase in the average reward value by using Method III, accompanied by a corresponding rise in the proportion of “expert experience” as training progresses. As the training progresses, the robot’s reliance on “expert experience” decreases and it starts to choose more autonomous planning paths. This leads to a slight decrease in average reward but an increase in stability. The robot model also begins to learn from “reverse experience,” resulting in an increase in average reward value and eventually obtaining navigation and obstacle avoidance abilities. With a 10× acceleration setting in Gazebo, 3500 cycles of training can be completed within just 4 h, the model convergence speed and training stability are significantly improved. The training model using Method III consistently yields high reward values throughout the entire process, with only a slight dip in the middle of training. Overall, the training is stable and this is demonstrated by both the kernel density plot (Figure 18) and bar hotspot chart (Figure 19) for Method III.

**Figure 20:**
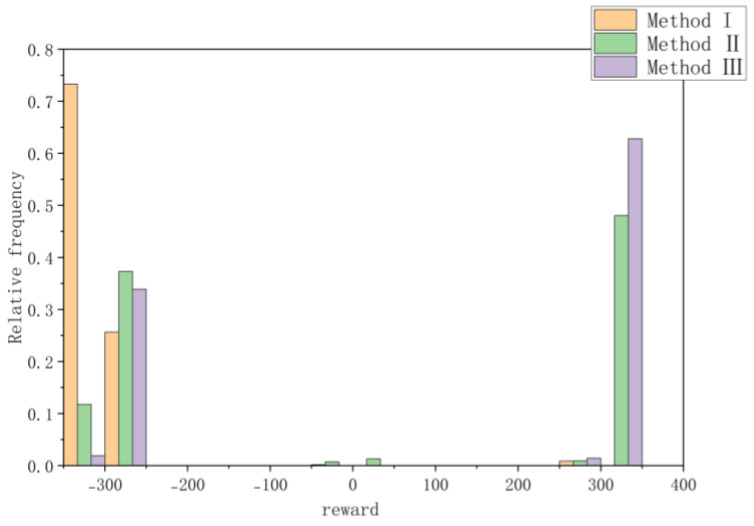
Relative Frequency Comparison of All Three Method.

Moreover, all three methods are compared in terms of data distribution histograms and numerical analysis, respectively. Figure 20 compares the reward level distributions for the three techniques. Compared to Method I, the reward values of Method II and Method III exhibit a higher degree of concentration around +300. Moreover, when compared to Method II, Method III exhibits a higher tendency to receive rewards valued at +300, resulting in a reward value distribution that is more closely aligned with this particular value.

Table 6 shows the results of the three kinds of numerical analysis, respectively, comparing the mean value, variance and quantile of the rewards obtained in the training process. Combined with Figure 18, the distribution and dispersion degree of the reward values obtained by the three different methods can be intuitively seen. Obviously, Method I has a low success rate and cannot converge. Compared with Method II, the average reward value of Method III is higher, and the standard deviation and variance are lower, indicating that the data concentration degree is higher, the model convergence speed is faster, and the stability is higher.

Therefore, the improved training methods proposed in this paper (Method II and Method III) exhibit superior performance compared to the traditional training method (Method I), including faster training time, higher stability and quicker model convergence.

**Figure 21:**
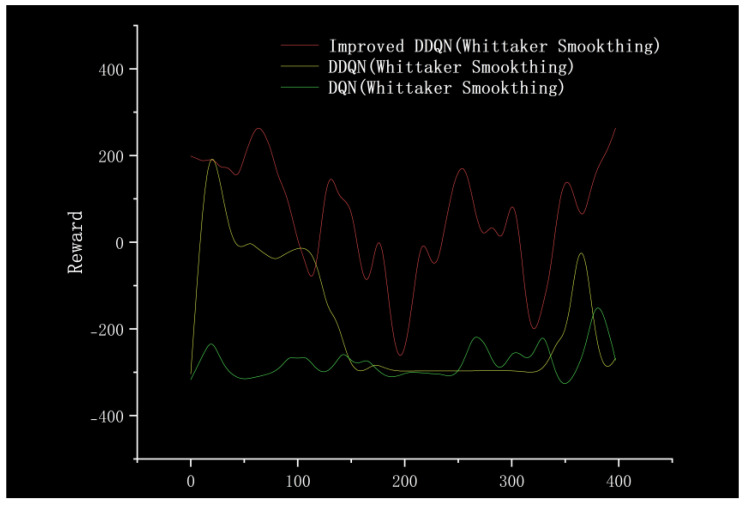
Comparison of the reward value.

**Figure 22:**
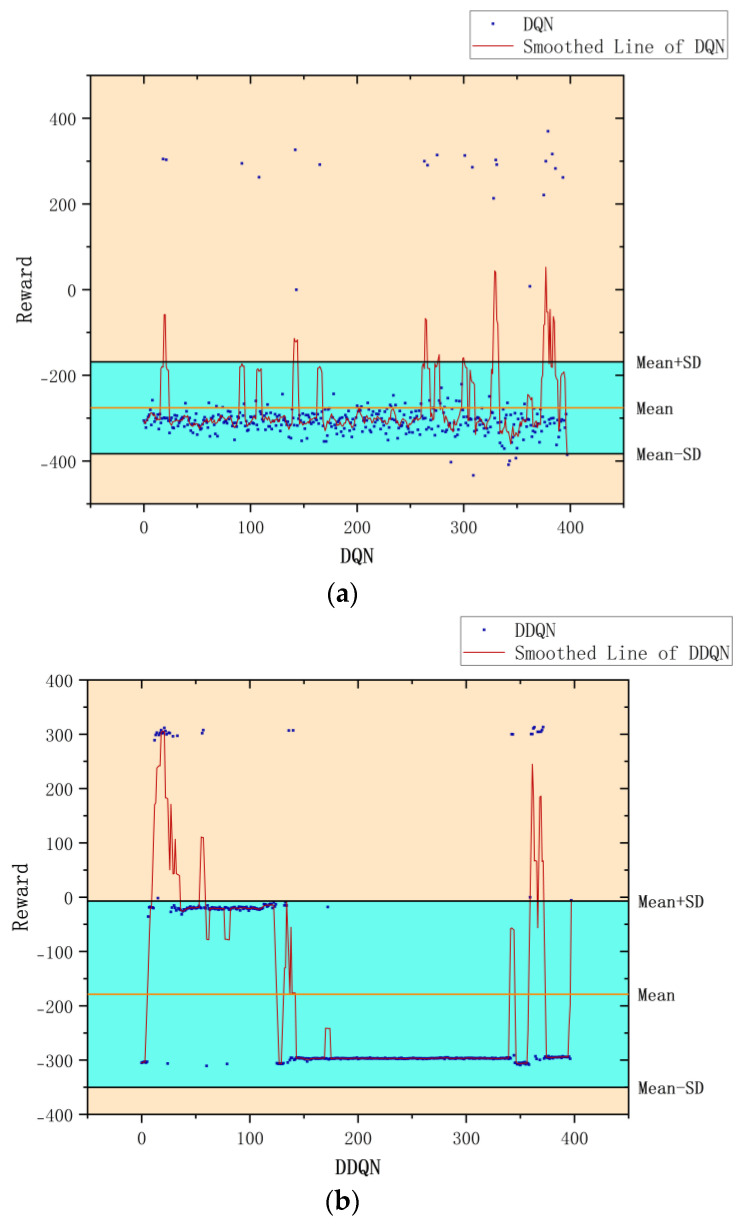

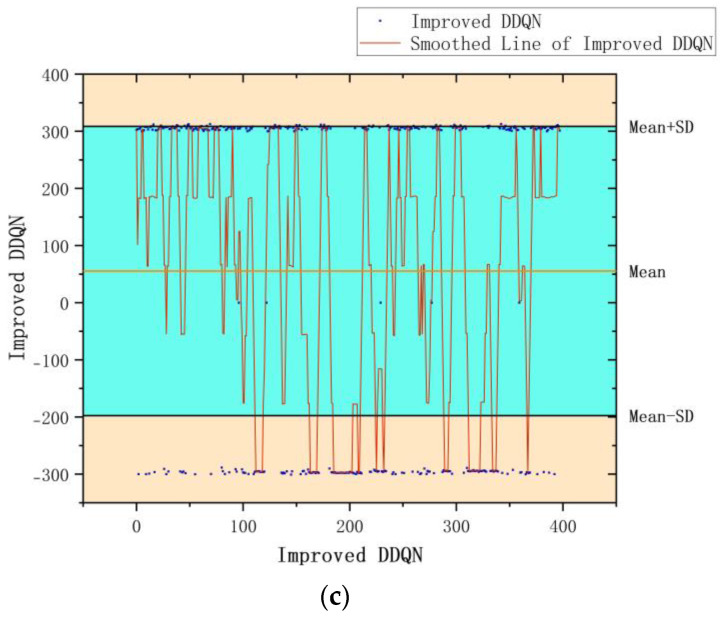
(**a**) The reward value obtained by using DQN. (**b**) The reward value obtained by using DDQN. (**c**) The reward value is obtained by using Improved DDQN.

In order to further verify the advantages of the improved algorithm proposed in this paper in the training process, this paper uses DQN, DDQN, and Improvement DDQN (Method III) for training under the same environment and analyzes the reward value obtained in each round, as shown in Figure 21 (using Whittaker Smoother fitting to create a smoother curve). We can see that the improved algorithm proposed in this paper results in more positive rewards in the training stage and higher overall rewards than the other two algorithms, as shown in Figure 22.

**Figure 23:**
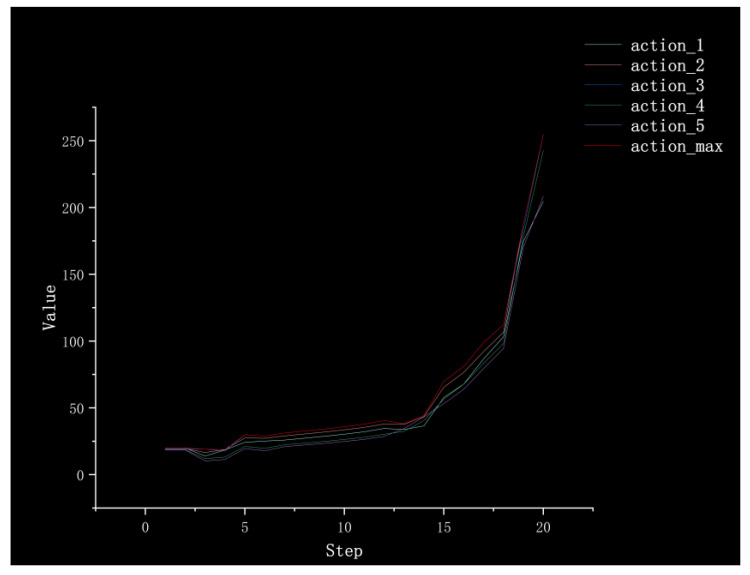
The Q value is obtained by each action taken during the path planning process.

In order to verify the feasibility of the improved algorithm and briefly analyze its interpretability, we used Method III to conduct a complete path planning process in the simulation environment and recorded the Q values of the actions that could be taken in each step, as shown in Figure 23. It can be seen that in the early stage of path planning, due to the farther distance from the target point, each Q value was relatively low. With the progress of the path finding process, at five steps and 13 steps, the overall reward is low due to the existence of obstacles around the robot, indicating that there may be dangers. Finally, after 20 steps, the target point is approaching, and the reward value becomes larger and larger until the target point is reached. The arrival of the target point will obtain a +300 reward; thus, it can be seen that taking different actions at the upcoming target point makes a big difference in the reward value.

**Figure 24:**
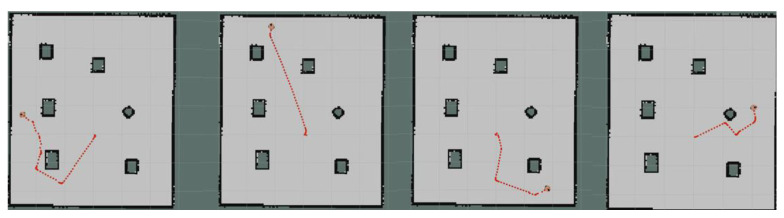
Results obtained by using Method II in a simple environment.

**Figure 25:**
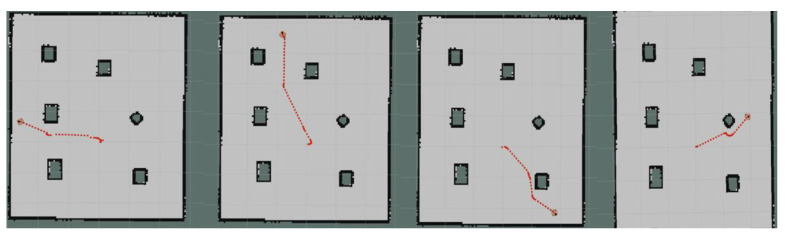
Results obtained by using Method III in a simple environment.

**Figure 26:**
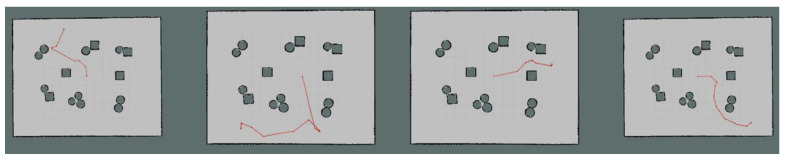
Results obtained by using Method II in a complex environment.

**Figure 27:**
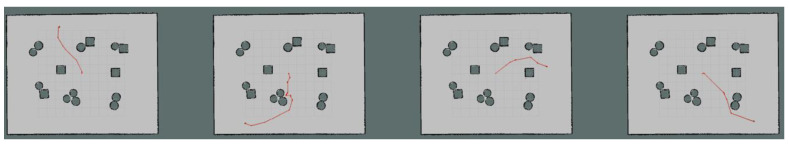
Results obtained by using Method III in a complex environment.

**Figure 28:**
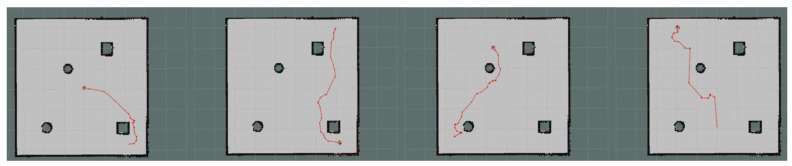
Results obtained by using Method II in different initial positions.

**Figure 29:**
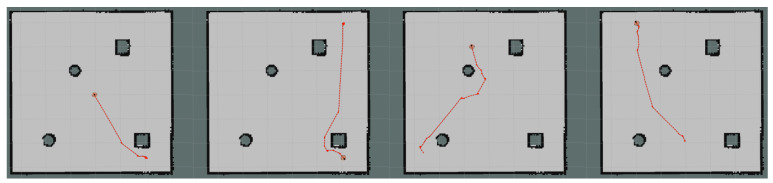
Results obtained by using Method III in different initial positions.

**Figure 30:**
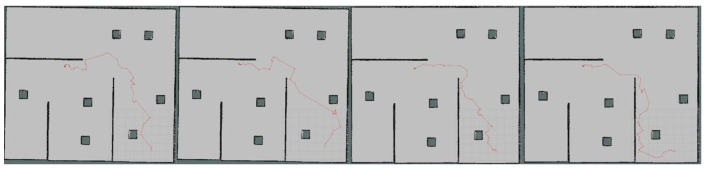
Multiple experiments using Method II on large-scale maps.

**Figure 31:**
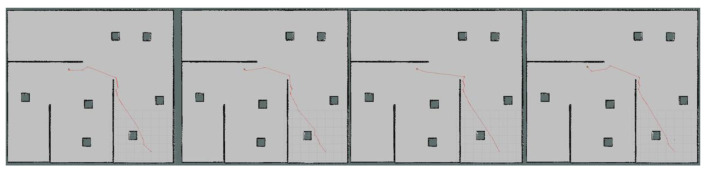
Multiple experiments using Method III on large-scale maps.

Moreover, we set up simple environment and complex environment, respectively, in the gazebo and carried out path planning by using models trained by different algorithms to evaluate the effect of the improved algorithm. Due to the poor stability, long time, and poor training effect of the training model using the traditional method, this paper only uses the improved algorithm for testing experiments. Figure 24 and Figure 25 show the trajectories obtained by using Method II and Method III for path planning of different target points, respectively, in a simple environment. It can be seen that the trajectories obtained using Method III are smoother, and the planned paths are shorter and more reasonable. Figure 26 and Figure 27 show the trajectories obtained by using Method II and Method III, respectively, for path planning of different target points in a complex environment. To eliminate the effect of different initial positions on the path planning, we use two methods to navigate at different initial positions separately, as shown in Figure 28 and Figure 29. We also conducted multiple experiments on the same path planning problem (with the same initial point and target point) using Method II and Method III on large-scale maps, as shown in Figure 30 and Figure 31. It can be seen that the trajectory obtained using Method III is better. Through simulation experiments, it can be seen that using the improvement proposed in this paper (Method III) can obtain a smoother path with a shorter distance.

### 4.3. Realistic Experiments

**Figure 32:**
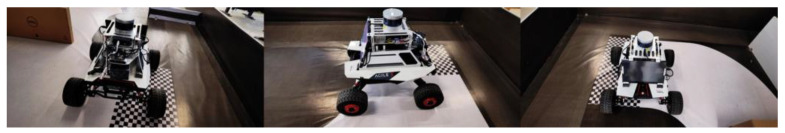
A mobile robot with LIDAR used in the experiment.

To verify the feasibility of the algorithm in a real environment, we use a real robot as shown in Figure 32 to execute the trained model-guided motion and we constructed a 5 m long and 5 m wide platform, equipped with randomly placed obstacles. After transferring the trained model from the simulation environment to the physical robot, we designed target point location information into the program. The robot (mobile robot) then executed corresponding actions based on instructions provided by the model. Since Method I of this paper is the most primitive method with only theoretical possibilities, the training process is very difficult and can easily become trapped in local optima, so only Method II and Method III are used for comparison in the real-world setting.

**Figure 33:**
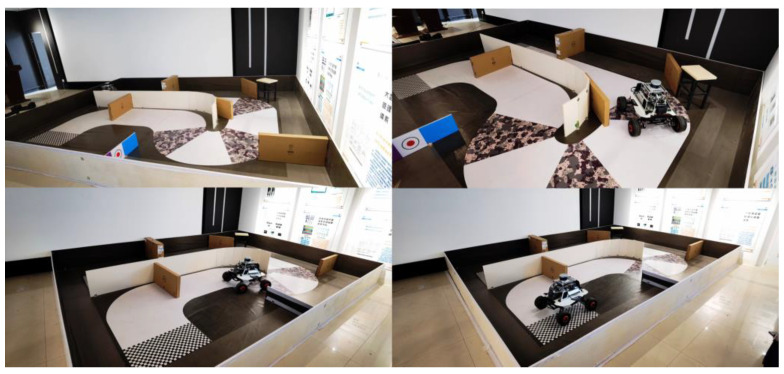
The robot is guided to move from the initial position to the specified position in the real environment.

As shown in Figure 33, the robot in our real-world experimental environment can move from the initial point to the target point on command with no collision and a smooth path. After conducting multiple experiments in a realistic environment, utilizing the model trained by Method III to guide robot actions, it was demonstrated that the proposed improved deep reinforcement learning path planning algorithm can efficiently and accurately construct collision-free paths.

## 5. Conclusions

We present an enhanced deep reinforcement learning approach for path planning in this paper. The proposed improved algorithm returns a specific action value that the robot should perform by receiving information from the LiDAR sensor. With these action values, the model will guide the robot to reach the target area while avoiding obstacles. First, the training data are discretized into a low-dimensional space. Second, we propose a two-branch network structure by segmenting the input from the navigation and obstacle-avoidance modules. Additionally, we introduce “expert experience” combining the Epsilon–Greedy algorithm with a prioritized experience replay strategy. Finally, we have improved the reward mechanism of traditional deep reinforcement learning to enable the robot to promptly receive feedback from the environment after each action. The experimental results in both real-world and virtual simulation scenarios that demonstrate that this enhanced approach accelerates model convergence, optimizes training stability and facilitates obstacle-free navigation.

In order to further enhance the effectiveness of reinforcement learning-based path planning in unknown environments, we intend to conduct subsequent research focusing on the following aspects:

1. Introduce the ICM module and Reverse Curriculum Learning module to explore more through curiosity mechanisms and design a reasonable step-by-step training process for distributed training of the model. It is believed that this will further improve the training speed and stability.
2. The dimensional discretization module is further improved to retain more information in the original data as far as possible under the premise of ensuring the speed and stability of the algorithm, so as to achieve better dimensional discretization.

## Figures and Tables

**Table 1:** The relative position of the target point to the robot.

Relative Location	Discrete Values
[−20, 20)	1
[20, 80)	2
[80, 180)	3
[180, −80)	4
[−80, −20)	5

**Table 2:** Discrete a continuous action to a lower dimensional space.

Value	Action	Linear Velocity	Angular Velocity
−2	Turn Right (big angle)	0.05	−1.5
−1	Turn Right (small angle)	0.1	−0.75
0	Go Straight	0.7	0
1	Turn Left (small angle)	0.1	0.75
2	Turn Left (big angle)	0.05	1.5

**Table 3:** Navigation Module Expert Experience.

Navigation Module Values	Expert Experience
(−20, 20)	0
(20, 80)	−1
(80, 180)	−2
(180, 280)	2
(280, 340)	1

**Table 4:** Obstacle Avoidance Module Expert Experience.

Obstacle Avoidance Module Value	Expert Experience
(L2, dis < 0.1)	−1
(L1, 0.5 < dis <= 0.1)	−1
(L1, dis < 0.1)	−2
(R1, dis < 0.1)	2
(R1, 0.5 < dis <= 0.1)	1
(R2, dis < 0.1)	1
Other	0

**Table 5:** Experimental parameters.

Parameters	Value
Memory Capacity	5000
α	0.8
Learning Rate	3 × 10^−5^
ε	0.99~0.05
β	0.4~1.0

**Table 6:** Data analysis of experimental results.

Parameters	Method I	Method II	Method III
Mean	−296.59856	10.83118	101.8214
Standard Deviation	56.62857	302.85006	289.28139
Lower 95% CI of Mean	−297.97611	2.84957	92.23436
Upper 95% CI of Mean	−295.221	18.81279	111.40845
Variance	3206.79515	91,718.15644	83,683.72419
1st Quartile (Q1)	−304.03949	−294.79913	−286.58769
Median	−301.76303	3.75054	309.77573
3rd Quartile (Q3)	−299.86398	317.04965	316.77985

## Data Availability

The data are not publicly available due to privacy.
